# Supplementary material for: Bi-directional ribosome scanning controls the stringency of start codon selection
Source: Nat Commun. 2021 Nov 15;12:6604. doi: 10.1038/s41467-021-26923-3 (PMC8593136; doi:10.1038/s41467-021-26923-3)
Supplement: Supplementary file 1 — Supplementary Information [file 41467_2021_26923_MOESM1_ESM.pdf]

SUPPLEMENTARY FIGURES

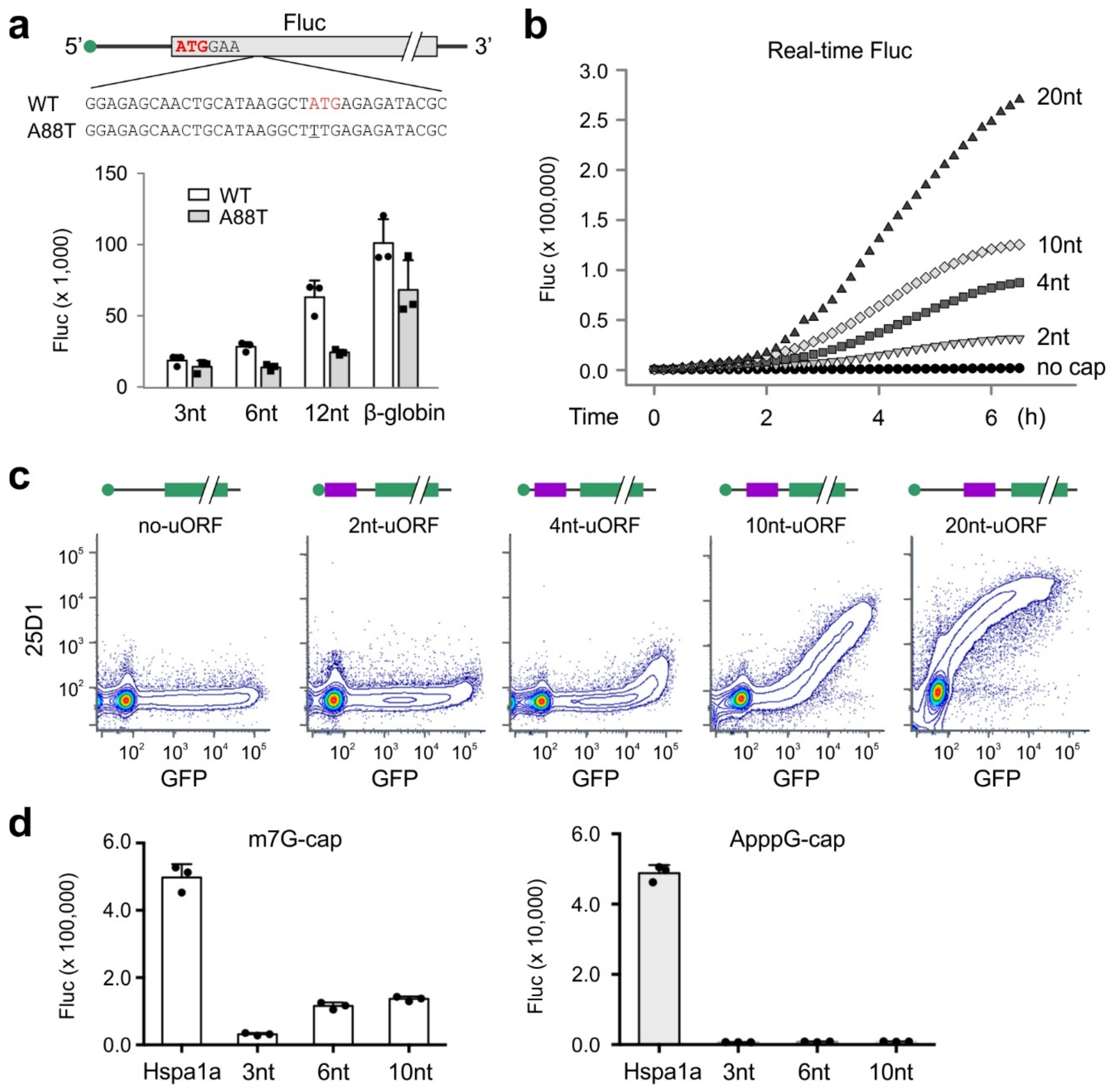

Supplementary Fig. 1. Characterization of Fluc mRNA reporters with ultra-short 5'UTR

a. The top panel shows the schematic of the wild type Fluc and the A88T mutant lacking the downstream AUG codon. The bottom panel shows the quantification of Fluc activities of WT or A88T mRNA reporters with varied 5'UTR length in transfected MEF cells. Error bars; mean ± SEM; n=3 biological replicates.

b. Representative real-time luminometry of MEF cells transfected with Fluc mRNA reporters bearing 5'UTR of the indicated length. Uncapped mRNA with 20-nt 5'UTR was included as negative control.

c. Representative flow cytometry scatterplots of HEK293-K^b cells transfected with plasmids of uORF reporters with 5'UTR of the indicated length. Plasmid with GFP only was included as negative control.

d. Fluc mRNA reporters capped with m^7G (the left panel) or the cap analog ApppG (the right panel) with varied 5'UTR length were transfected into MEF cells followed by quantification of Fluc activities. Error bars; mean ± SEM; n=3 biological replicates.

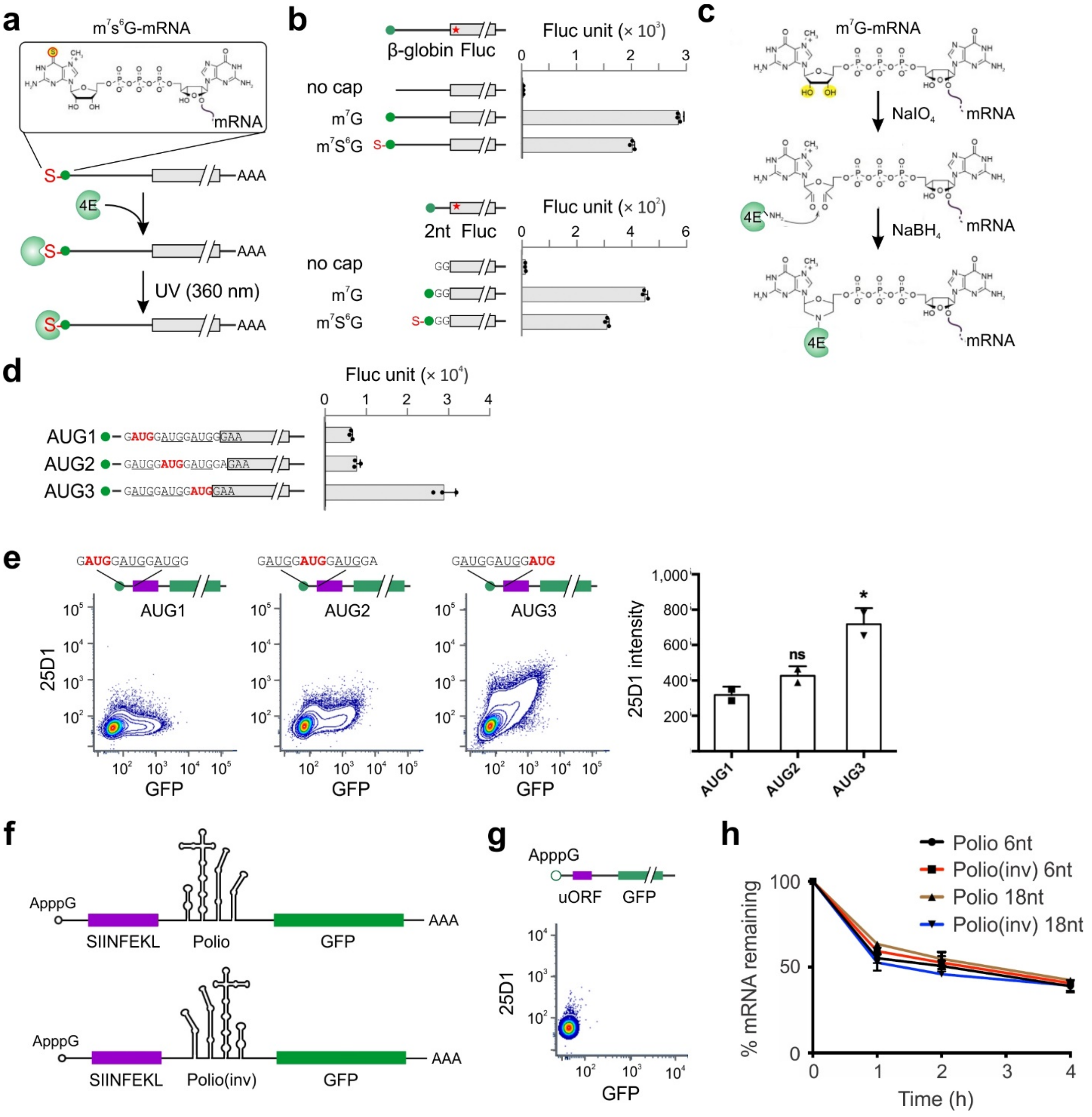

Supplementary Fig. 2. Examine bi-directional scanning of PIC using mRNA reporters

a. Schematic of cross-linking between m^7s^6G capped mRNAs with purified eIF4E under UV 360 nm. In vitro synthesized mRNAs were capped with 6-Thio-GTP using capping enzyme system. m^7s^6G capped mRNAs were incubated with purified eIF4E in the binding buffer at 4°C under 360 nm UV exposure for 15 min.

b. Characterization of m^7s^6G capped mRNA reporters. Uncapped mRNA, m^7G or m^7s^6G capped mRNAs with either β-globin (upper) or 2-nt (bottom) 5'UTR were incubated with

RRL. Fluc activities were monitored by luminometry. Error bars; mean ± SEM; n=3 biological replicates.

c. Schematic of cross-linking between m^7G capped mRNAs with purified eIF4E via redox reaction. m^7G capped mRNA are oxidized in presence of sodium periodate, followed by ethanol precipitation. Oxidized mRNA was incubated with purified eIF4E in the binding buffer at 4°C for 15 min. Sodium borohydride was added to the mixtures, followed by incubation for 2-3 hr on ice.

d. Fluc mRNA reporters containing 3 AUG codons near the 5' end were transfected into MEF cells, followed by quantification of Fluc activities by real-time luminometry. Error bars; mean ± SEM; n=3 biological replicates.

e. Representative flow cytometry scatterplots of HEK293-K^b cells transfected with uORF reporters containing 3 AUG codons near the 5'end. AUG1 (left), AUG2 (middle) or AUG3 (right) is in the same fame of uORF encoding SIINFEKL (purple). The right panel shows the quantification of GFP and 25D1 fluorescence intensity from transfected HEK293-K^b cells. Error bars: mean ± SEM; $n = 3$ biological replicates. Two-way ANOVA, * $p < 0.05$.

f. Schematic of the uORF reporters with the polio IRES inserted between uORF and GFP. The bottom panel shows the same reporter with an inversed IRES element.

g. Representative flow cytometry scatterplots of HEK 293-K^b cells transfected with uORF reporters with neither m7G cap nor the IRES element.

h. HEK293 cells were transfected with indicated mRNA reporters followed by RT-qPCR at indicated time points. n = 3 biological replicates; Error bars indicate SEM.

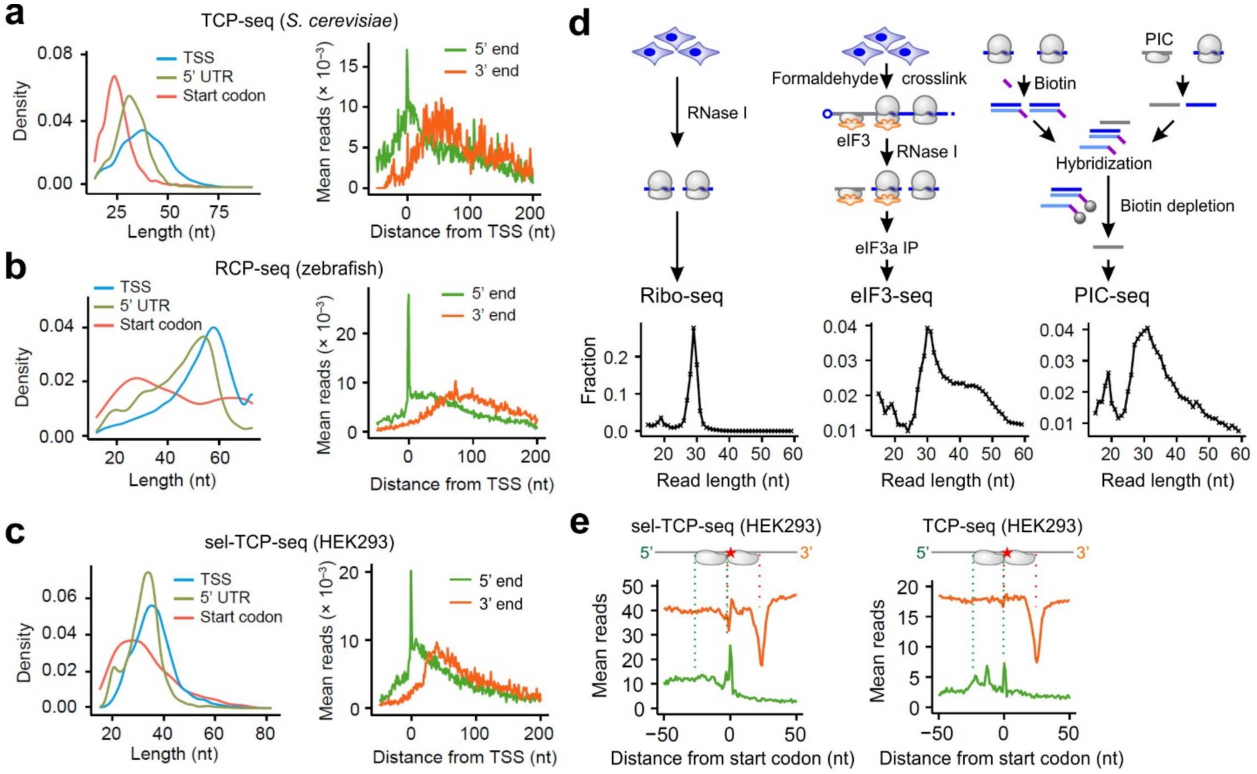

Supplementary Fig. 3. Characterizing PIC footprints

- **a.** Reanalysis of TCP-seq data sets obtained from *S. cerevisiae* (Archer et al). The left panel shows the length distribution of reads mapped to TSS, 5'UTR, or near start codons. The right panel shows the aggregation plots of 5' end (green) and 3' end (red) of reads on mRNAs aligned to TSS. Two-sided Wilcoxon test, all P values $< 2.2\times10^{-16}$.
- **b.** Reanalysis of RCP-seq data sets obtained from zebrafish (Giess et al). Two-sided Wilcoxon test, all P values $< 2.2\times10^{-16}$.
- **c.** Reanalysis of sel-TCP-seq data sets obtained from HEK293T cells (Wagner et al). Two-sided Wilcoxon test, P values of TSS and 5'UTR $< 2.2\times10^{-16}$, the P value of start codon = 1.4×10^{-7}.
- **d.** Schematic of Ribo-seq, eIF3-seq, and PIC-seq. For Ribo-seq, polysomes were separated from whole cell lysates using sucrose gradients. Collected polysome fractions were digested with RNase I followed by deep sequencing of ribosome-protected mRNA fragments. For eIF3-seq, cells were fixed by formaldehyde followed by polysome separation and RNase I digestion. Collected 40S and 80S fractions were subjected to immunoprecipitation using anti-eIF3a antibodies. Purified eIF3-associated ribosome footprints were subjected to deep sequencing. For PIC-seq, cells were fixed by formaldehyde followed by polysome separation and RNase I digestion. Purified ribosome footprints from 80S fractions were reverse transcribed using biotinylated primer. The biotin labeled cDNA was hybridized to the purified footprints from 40S fractions. After depletion by streptavidin beads, the PIC-associated ribosome footprints were enriched and subjected to deep sequencing. The bottom panels are read length distribution for 5'UTR reads obtained from Ribo-seq, eIF3-seq and PIC-seq.

e. Characterization of PIC footprints from sel-TCP-seq and TCP-seq obtained from HEK293T cells (Wagner et al). Both the 5′ end (green) and 3′ end (red) of reads were mapped to transcripts aligned to annotated start codons (red star).

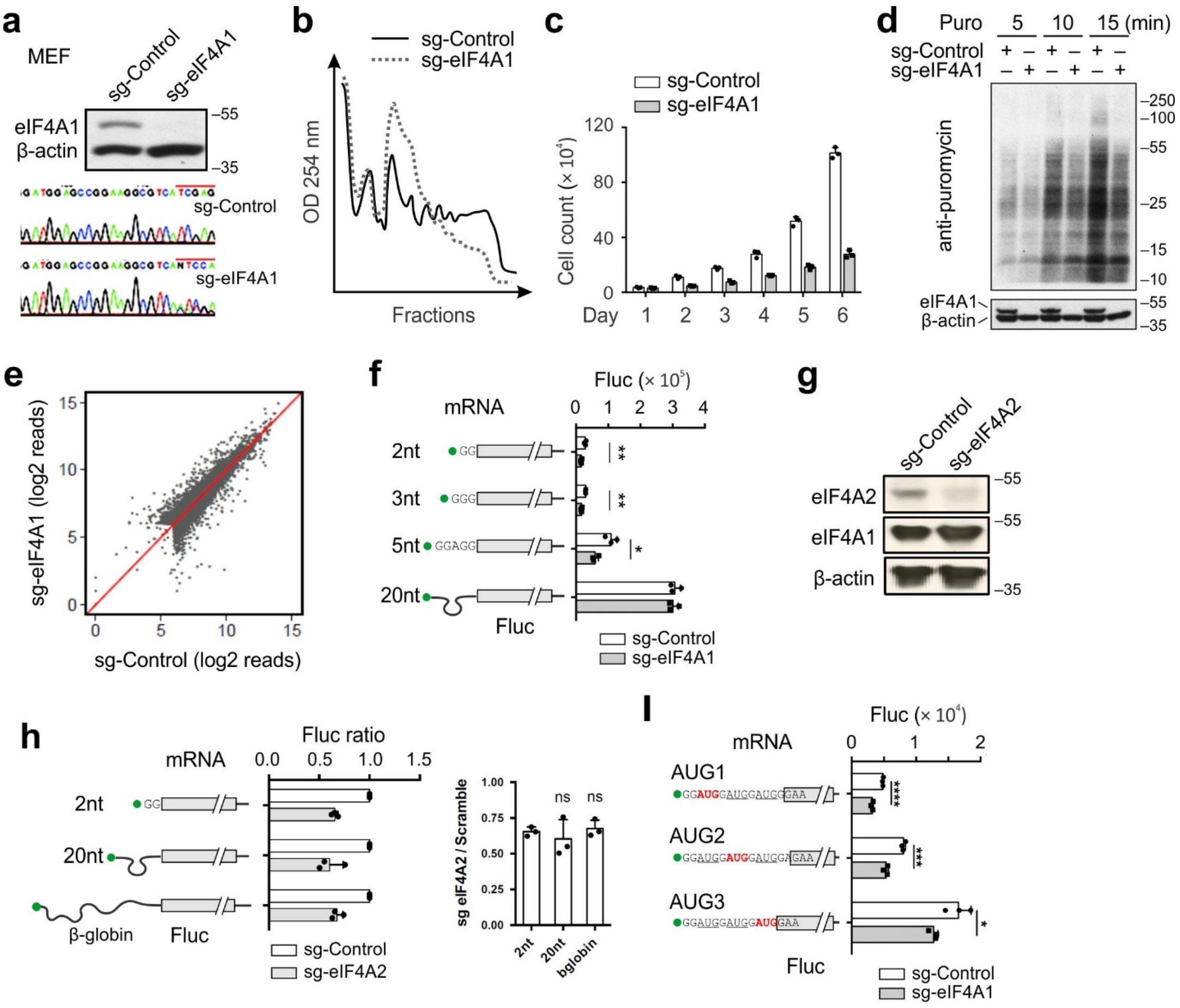

Supplementary Fig. 4. Characterization of MEF cells with eIF4A1 knockdown

a. Immunoblotting of MEF cells with or without eIF4A1 knockdown. Representative results of three independent experiments are shown.

b. Polysome profiles of MEF cells with or without eIF4A1 knockdown were analyzed by sucrose density gradient centrifugation in polysome buffer.

c. Proliferation rates of MEF cells with or without eIF4A1 knockdown. Cell numbers were normalized to the value obtained on Day 1. Error bars: mean ± SEM; n = 3 biological replicates.

d. Nascent proteins in MEF cells with or without eIF4A1 knockdown were labeled with 10 µg/ml of puromycin for 5, 10 or 15 mins. Cell lysates were resolved by SDS-PAGE and analyzed by immunoblotting using the indicated antibodies. Representative results of three independent experiments are shown.

e. Using Ribo-seq data sets, a scatter plot shows the correlation of individual CDS ribosome occupancy between MEF cells with or without eIF4A1 knockdown.

f. Translation efficiency of Fluc mRNA reporters with ultra-short 5'UTR in MEF cells with or without eIF4A1 knockdown. Error bars: mean ± SEM; $n = 3$ biological replicates. Two-tailed t-test, * $p < 0.05$; *** $p < 0.001$.

g. Immunoblotting of MEF cells with or without eIF4A2 knockdown. Representative results of two independent experiments are shown.

h. Translational efficiency of Fluc mRNA reporters with different 5'UTR in MEF cells with or without eIF4A2 knockdown. The right panel shows the ratio of Fluc levels before and after eIF4A2 knockdown. Error bars; mean ± SEM; n=3 biological replicates. Two-way ANOVA, ns, not significant.

i. Translation efficiency of Fluc mRNA reporters containing 3 AUG codons near the 5'end in MEF cells with or without eIF4A1 knockdown. Error bars: mean ± SEM; $n = 3$ biological replicates. Two-tailed t-test, * $p < 0.05$; *** $p < 0.001$; **** $p < 0.0001$.

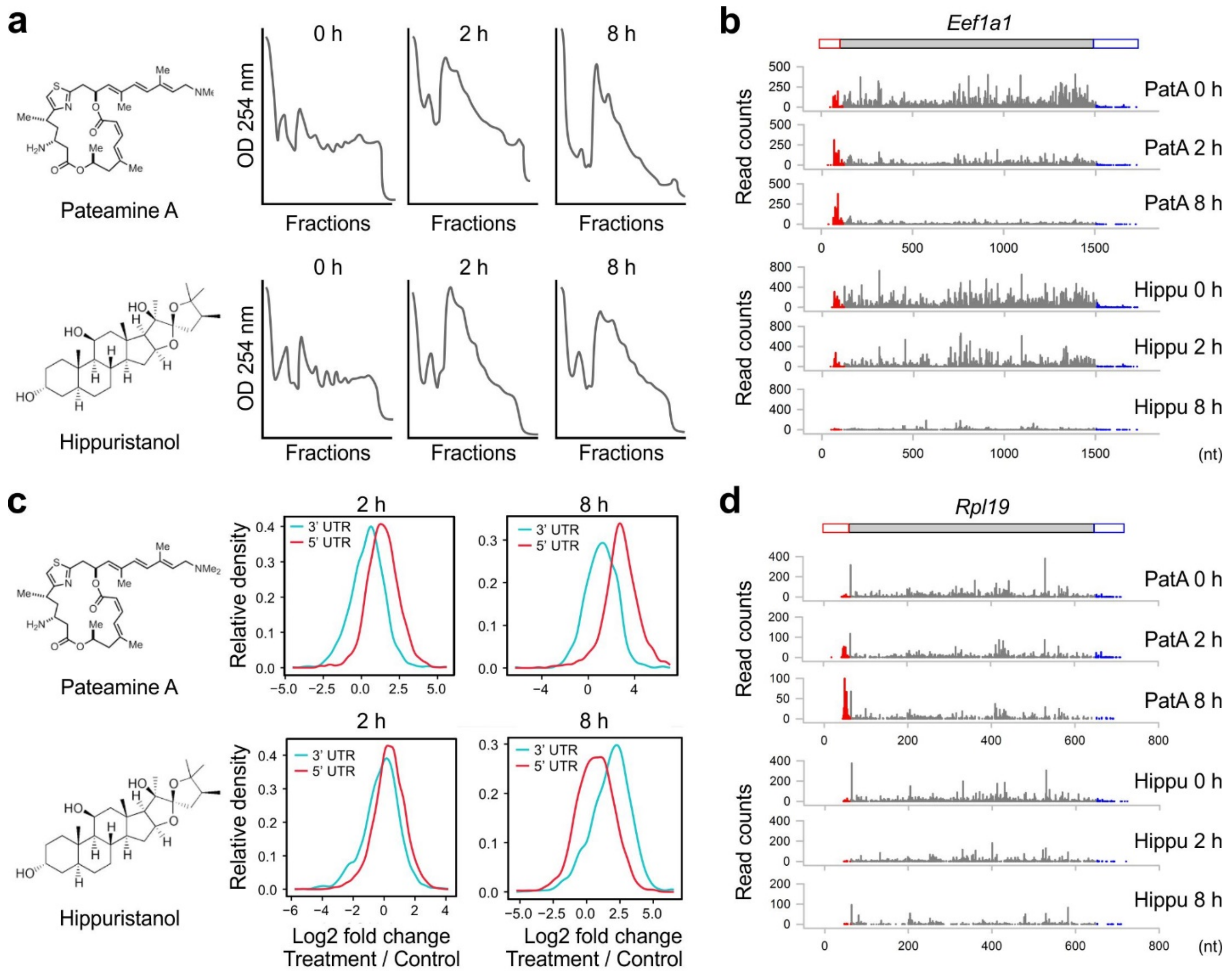

Supplementary Fig. 5. Enhanced the ATPase activity of eIF4A1 promotes alternative translation in 5'UTR

a. Polysome profiles of MEF cells treated with eIF4A1 modulators. The upper panels show the polysome profiling of MEF cells treated with 50 nM PatA for different times. The bottom panels show polysome profiling of MEF cells treated with 200 nM Hippu for different times.

b. A representative example of genes (*Eef1a1*) shows differential ribosome occupancy in 5'UTR after treatment with eIF4A1 modulators.

c. Distribution of fold changes for 5'UTR ribosome occupancy after treatment with eIF4A1 modulators (red line). 3'UTR read density was used as internal controls (blue line). Two-sided Wilcoxon test, all *P* values $< 2.2\times10^{-16}$.

d. A representative example of genes (*Rpl19*) shows newly emerged ribosome occupancy in 5'UTR after treatment with PatA, but not Hippu.
