# Supplementary material for: Bi-directional ribosome scanning controls the stringency of start codon selection
Source: Nat Commun. 2021 Nov 15;12:6604. doi: 10.1038/s41467-021-26923-3 (PMC8593136; doi:10.1038/s41467-021-26923-3)
Supplement: Supplementary file 3 — Description of Additional Supplementary Files [file 41467_2021_26923_MOESM3_ESM.pdf]

Description of Additional Supplementary Files

File Name: Supplementary Data 1
Description: List of primers and sgRNA targeting sequences
