# Supplementary material for: Bi-directional ribosome scanning controls the stringency of start codon selection
Source: Nat Commun. 2021 Nov 15;12:6604. doi: 10.1038/s41467-021-26923-3 (PMC8593136; doi:10.1038/s41467-021-26923-3)
Supplement: Supplementary file 5 — Reporting Summary [file 41467_2021_26923_MOESM5_ESM.pdf]

nature research

Corresponding author(s): Shu-Bing Qian

Last updated by author(s): Oct 23, 2021

Reporting Summary

Nature Research wishes to improve the reproducibility of the work that we publish. This form provides structure for consistency and transparency in reporting. For further information on Nature Research policies, see our Editorial Policies and the Editorial Policy Checklist.

Statistics

For all statistical analyses, confirm that the following items are present in the figure legend, table legend, main text, or Methods section.

n/a	Confirmed	
☐	☒	The exact sample size (*n*) for each experimental group/condition, given as a discrete number and unit of measurement
☐	☒	A statement on whether measurements were taken from distinct samples or whether the same sample was measured repeatedly
☐	☒	The statistical test(s) used AND whether they are one- or two-sided<br>*Only common tests should be described solely by name; describe more complex techniques in the Methods section.*
☒	☐	A description of all covariates tested
☒	☐	A description of any assumptions or corrections, such as tests of normality and adjustment for multiple comparisons
☐	☒	A full description of the statistical parameters including central tendency (e.g. means) or other basic estimates (e.g. regression coefficient) AND variation (e.g. standard deviation) or associated estimates of uncertainty (e.g. confidence intervals)
☐	☒	For null hypothesis testing, the test statistic (e.g. *F*, *t*, *r*) with confidence intervals, effect sizes, degrees of freedom and *P* value noted<br>*Give P values as exact values whenever suitable.*
☒	☐	For Bayesian analysis, information on the choice of priors and Markov chain Monte Carlo settings
☒	☐	For hierarchical and complex designs, identification of the appropriate level for tests and full reporting of outcomes
☒	☐	Estimates of effect sizes (e.g. Cohen's *d*, Pearson's *r*), indicating how they were calculated

Our web collection on statistics for biologists contains articles on many of the points above.

Software and code

Policy information about availability of computer code

Data collection	Firefly luciferase activities were monitored and recorded using Kronos Dio Luminometer (Atto). qPCR assays were carried on a LightCycler 480 Real-Time PCR System (Roche Applied Science). Immunoblots were performed using enhanced chemiluminescence (ECL plus, GE Healthcare). Flow cytometry was performed on a BD FACSAria Fusion flow cytometer (BD Biosciences). The primer extension was run on an ABI 3730xl instrument. High through-put sequencing data was collected by illumina HiSeq Control Software v2.2.58 for HiSeq2500 System.
Data analysis	FCS Express (Version 6) and FlowJo were used to analyze cytometry data. Prism (Version 6) was used to analyze data, generate decay graphs and standard bar graphs. Peak Scanner 2 software was used to analyze toe-printing data.<br>Cutadapt v1.18 was used to filter low quality reads. R v3.5.1 was used to perform all statistical analysis. ggplot2 for R was used to make all statistical figures. MEME v4.11.2 was used to motif analysis. The custom Perl scripts was used for all other data analyses, which are available on request to the corresponding authors.

For manuscripts utilizing custom algorithms or software that are central to the research but not yet described in published literature, software must be made available to editors and reviewers. We strongly encourage code deposition in a community repository (e.g. GitHub). See the Nature Research guidelines for submitting code & software for further information.

Data

Policy information about availability of data

All manuscripts must include a data availability statement. This statement should provide the following information, where applicable:

- Accession codes, unique identifiers, or web links for publicly available datasets
- A list of figures that have associated raw data
- A description of any restrictions on data availability

All sequencing data have been deposited to GEO database. All other data supporting the findings of this study are available from the corresponding authors on

reasonable request.

Field-specific reporting

Please select the one below that is the best fit for your research. If you are not sure, read the appropriate sections before making your selection.

- [x] Life sciences
- [ ] Behavioural & social sciences
- [ ] Ecological, evolutionary & environmental sciences

For a reference copy of the document with all sections, see nature.com/documents/nr-reporting-summary-flat.pdf

Life sciences study design

All studies must disclose on these points even when the disclosure is negative.

Sample size	No sample size was predetermined. Three or more independent replicates were done as three replicates are sufficient for inferential statistical analysis. All sample sizes and the number of replicates were stated in figure legends.
Data exclusions	No data were excluded from the analyses.
Replication	Experimental findings were reliably reproduced. Replication were described in figure legends.
Randomization	All experiments were performed on cell lines, and no animal studies were performed. Cells from each cell line have the same genetic background and phenotype, hence no randomization was used.
Blinding	Investigators were blinded to group allocation during data collection and analysis.

Reporting for specific materials, systems and methods

We require information from authors about some types of materials, experimental systems and methods used in many studies. Here, indicate whether each material, system or method listed is relevant to your study. If you are not sure if a list item applies to your research, read the appropriate section before selecting a response.

Materials & experimental systems

n/a	Involved in the study	
[ ]	[x]	Antibodies
[ ]	[x]	Eukaryotic cell lines
[x]	[ ]	Palaeontology and archaeology
[x]	[ ]	Animals and other organisms
[x]	[ ]	Human research participants
[x]	[ ]	Clinical data
[x]	[ ]	Dual use research of concern

Methods

n/a	Involved in the study	
[x]	[ ]	ChIP-seq
[ ]	[x]	Flow cytometry
[x]	[ ]	MRI-based neuroimaging

Antibodies

Antibodies used	Anti-eIF4A1 (Abcam, ab31217), used at 1:1000;<br>Anti-eIF4A2 (Life technologies, PA585142), used at 1:1000;<br>Anti-puromycin (Developmental Studies Hybridoma Bank, PMY-2A4), used at 1:1000;<br>Anti-β-Actin (Sigma, A5441), used at 1:5000;<br>Anti-eIF3a antibody (Cell signaling, 3411S), used at 1:1000<br>Goat anti-rabbit Alexa Fluor 594 (Invitrogen, A-11007), used at 1:2000;<br>Goat anti-mouse Alexa Fluor 546 (Invitrogen, A-10036), used at 1:2000;<br>25D1 monoclonal antibody (from J. W. Yewdell lab, see ref. 28).
Validation	Antibodies were validated as noted on manufacturer's website and were independently tested/verified by the authors as follows:<br>Anti-eIF4A1 (Abcam, ab31217): Company website application listed: WB, IP, ICC/IF, IHC in H M R C Mk; Application tested in the manuscript: WB (1:1000) in human and mouse<br>Anti-eIF4A2 (Life technologies, PA585142): Company website application listed: WB in Human, Mouse (100%), Rat (100%), Zebrafish (91%), Pig (100%), Chicken (95%), Rhesus Monkey (100%), Chimpanzee (100%), Bovine (100%); Application tested in the manuscript: WB (1:1000) in human and mouse<br>Anti-puromycin (Developmental Studies Hybridoma Bank, PMY-2A4): Company website application listed: WB, IP, ELISA, FACS in all; Application tested in the manuscript: WB (1:1000) in mouse<br>Anti-β-Actin (Sigma, A5441): Company website application listed: WB, ELISA, IF, IHC in pig, Hirudo medicinalis, bovine, rat, canine, feline, human, rabbit, carp, mouse, guinea pig, chicken, sheep; Application tested in the manuscript: WB (1:5000) in human and mouse<br>Anti-eIF3a antibody (Cell signaling, 3411S): Company website application listed: WB, IP, IF, IHC in H M R Mk; Application tested in the

manuscript: WB (1:1000), IP (1:50) in human and mouse

Eukaryotic cell lines

Policy information about cell lines

Cell line source(s)	MEF cells (mouse, provided by the Laboratory of David J. Kwiatkowski)<br>HEK293 cells (human, purchased from the American Type Culture Collection (ATCC))<br>HEK293-Kb cells (human, provided by J. W. Yewdell lab)<br>Lenti-X 293T cells (Clontech)
Authentication	Cell lines were not authenticated by ourselves.
Mycoplasma contamination	All cell lines tested negative for mycoplasma contamination prior to our studies.
Commonly misidentified lines (See ICLAC register)	No commonly misidentified cell lines were used.

Flow Cytometry

Plots

Confirm that:

- [x] The axis labels state the marker and fluorochrome used (e.g. CD4-FITC).
- [x] The axis scales are clearly visible. Include numbers along axes only for bottom left plot of group (a 'group' is an analysis of identical markers).
- [x] All plots are contour plots with outliers or pseudocolor plots.
- [x] A numerical value for number of cells or percentage (with statistics) is provided.

Methodology

Sample preparation	Transfected cells are washed with PBS and harvested by trypsin. Cells are then re-suspended in blocking buffer (1% bovine serum albumin (BSA) in PBS). Cells are aliquoted into a 96-well plate followed by 2000 rpm spinning for 2 min. After removal of blocking buffer, cells are washed one more time followed by staining with antibody. After incubation in the dark with gentle rocking at 4°C for 30 minutes, cells are washed three times with 200 uL of the blocking buffer to remove unbound antibodies. Resuspend cells in 300 uL of blocking buffer followed by single cell filtering (Falcon). Cells are analyzed on a BD FACSAria Fusion flow cytometer (BD Biosciences). Cytometry data analysis is conducted using FCS Express.
Instrument	BD FACSAria Fusion flow cytometer.
Software	FCS Express (Version 6).
Cell population abundance	At least 10,000 counts were recorded using a 0.5 mL s-1 flow rate.
Gating strategy	Cells were first assessed in the FSC-A/SSC-A dot plot to exclude cell debris (P1) and doublet discrimination was carried out by additional plots to remove doublets and cell clumps (P2 and P3). Finally, fluorescence was detected in the FITC, APC or PE channel. For a negative control, cells are transfected with Lipofectamine MessengerMAX only. Tools for fluorescence compensation were applied whenever needed. A detailed description of the experimental design can be found in the main manuscript.

- [x] Tick this box to confirm that a figure exemplifying the gating strategy is provided in the Supplementary Information.
